# Effect of delayed CNI-based immunosuppression with Advagraf® on liver function after MELD-based liver transplantation [IMUTECT]

**DOI:** 10.1186/1471-2482-14-64

**Published:** 2014-09-01

**Authors:** Susanne Richter, Georg Polychronidis, Daniel N Gotthardt, Philipp Houben, Thomas Giese, Anja Sander, Colette Dörr-Harim, Markus K Diener, Peter Schemmer

**Affiliations:** 1 Department of General, Visceral and Transplant Surgery, Heidelberg, Germany; 2 Department of Internal Medicine IV, University of Heidelberg, Heidelberg, Germany; 3 Immunology, University of Heidelberg, Heidelberg, Germany; 4 Institute of Medical Biometry and Informatics, University of Heidelberg, Heidelberg, Germany; 5 Study Centre of the German Surgical Society (SDGC), Heidelberg, Germany

**Keywords:** Advagraf®, Prospective observational study, MELD-score, Liver function, LiMAx test, Infection rate, HLA-DR status, Immunostatus

## Abstract

**Background:**

MELD-based allocation for liver transplantation follows the “sickest-patient-first” strategy. The latter patients present with both, decreased immune competence and poor kidney function which is further impaired by immunosuppressants.

**Methods/Design:**

In this prospective observational study, 50 patients with de novo low-dose standard Advagraf®-based immunosuppression consisting of Advagraf®, Mycophenolat-mofetil and Corticosteroids after liver transplantation will be evaluated. Advagraf® trough levels of 7-10 μg/l will be reached at the end of the first postoperative week. Immunostatus, infectious complications, graft and kidney function are compared between patients with a pretransplant calculated MELD-score of ≤20 and >20. Each group comprises of 25 consecutive patients. Prior to liver transplantation and on the postoperative days 1, 3 and 7, the patients’ graft function (LiMAx test) will be evaluated. On the postoperative days 3, 5 and 7 the patients’ immune status will be evaluated by the measurement of their monocytic HLA-DR status.

Infectious complications (CMV-reactivation, wound infection, urinary tract infection, and pneumonia), graft- and kidney function will be analysed on day 0, within the first week, and 1, 3, 6, 9 and 12 months after liver transplantation.

**Discussion:**

This study was designed to assess the effect of a standard low-dose Calcineurin inhibitor-based immunosuppression regime with Advagraf® on the rate of infectious complications, graft and renal function after liver transplantation.

**Trial registration:**

The trial is registered at “Clinical Trials” (http://www.clinicaltrials.gov), NCT01781195.

## Background

Liver transplantation (LT) is the only curative treatment for patients with decompensated liver cirrhosis. The model for end-stage liver disease score (MELD-score) is used for the prioritization of patients awaiting LT [1].

The MELD-score was initially developed to evaluate the prognosis after transjugular intrahepatic porto-systemic shunt (TIPS). The laboratory parameters creatinine, bilirubin, and international normalized ratio (INR) are needed for calculation of this score [1]. Nowadays, the MELD-score is also used to allocate grafts for LT. The calculated MELD-scores of patients waiting for LT usually range between 6 and 40 [2].

Sawitzki *et al.* described markers that could be used to determine a patient’s immune status. This would make it possible to tailor the extent of the individual’s immunosuppression therapy after LT. However, the lowest possible dose of immunosuppressant needs to be determined individually in order to minimize side effects of immunosuppression while also avoiding an increase in the rejection rate [3]. The patient’s immune status can be evaluated by the measurement of B-cell sensitization and soluble biomarkers like CD30 [4,5]. In addition, the immune status after LT, sepsis, injuries, and surgery can be evaluated by the monocytic HLA-DR status. A decreased monocytic HLA-DR status is indicative of an impaired immune competence [6].

In this context, it would be interesting to investigate to what degree a patient’s HLA-DR status corresponds to the labMELD-/Na-MELD-score and to measure the liver function with the LiMAx test. Furthermore, this trial aims to assess whether or not high-MELD patients (MELD-score >20) can benefit from a low-dose, prolonged-release immunosuppression with Advagraf®.

HLA-DR belongs to the major histocompatibility complex (MHC)-II family and is important for antigen presentation to CD4 + -T-cells [6,7]. Monocytes express HLA-DR on their surface, which can easily be detected by flow cytometry [6]. As mentioned above, the monocytic HLA-DR status has been used to evaluate the immune status after surgery, trauma, or during sepsis [6]. Moreover, the decreased monocytic function found during sepsis may be due to a diminished HLA-DR expression [8].

Granulocyte-Macrophage Colony-Stimulating-Factor (GM-CSF) activates monocytes and prevents apoptosis, in addition, GM-CSF increases monocytic HLA-DR expression [8]. Havemann *et al.* measured the monocytic HLA-DR status after LT in 20 patients and reported that a decreased monocytic HLA-DR status was associated with the risk of sepsis after LT. The risk of sepsis increases with the administration of an elevated Prednisolone dosage [7,9]. Administering reduced doses of Prednisolone may prevent patients with decreased monocytic HLA-DR statuses from becoming septic [7].

In order to allow for multi-center trials on the monocytic HLA-DR status and in an effort to index a patient’s immune status, a standardized BD Quantibrite™ HLA-DR/monocyte reagent was developed by Döcke *et al.*[6]. For consistent results, the blood should be stored on ice in EDTA tubes and must be analyzed within 4 hours after extraction [6].

The LiMAx test is useful for pre- and postoperative examinations of patients undergoing extensive liver resections to determine both, the risk of postoperative liver failure and liver function. The same applies to patients after LT [10]. The LiMAx test is usually done at the patient’s bedside. Patients are required to lay in bed and avoid eating or smoking within 3 hours prior to being given ^13^C-Methacetin infusion. The enzyme CYP1A2 is exclusive for hepatocytes and metabolizes ^13^C-Methacetin to Paracetamol and ^13^CO_2_. Thus the ^13^CO_2_/^12^CO_2_ ratio in the exhaled air correlates with liver function [11].

Unlike the LiMAX test, the sole use of laboratory parameters (e.g. bilirubin, ammonia, INR, and glutamatdehydrogenase) is not optimal for analyzing patients’ graft function, since bilirubin levels are dependent on preoperative levels and indicate transplant failure with a delay of a few days. An increased INR can also be found as a consequence of bleeding, disseminated intravascular coagulation (DIC) or as a side effect of several medications [10]. Early recognition of graft dysfunction is necessary as hepatic dysfunction affects metabolism and elimination of Tacrolimus with the need of future dose adjustments [12].

Since the MELD-score is often used for graft allocation before LT and the underlying laboratory values are dependent on the methods used at the time of measurement, it is possible for the MELD-scores to vary significantly among the different laboratories [13,14]. It would be interesting to investigate the correlation between the pre-operative MELD-score and the LiMAx results, and determine whether or not this correlation has any influence on the patient’s preoperative immunostatus (HLA-DR status).

At our institution, a Tacrolimus-based immunosuppression (Advagraf®, Prograf®) belongs to the standard immunosuppression for patients undergoing LT.

Negative side effects of Calcineurin inhibitors (CNI) are, among others, renal impairment and infectious complications [15]. High-MELD patients often present with a limited renal function as creatinine is one component of the MELD-score [1]. The combination of CNI application and higher MELD-scores increases the risk of renal impairment [15]. Furthermore it has been hypothesized that high-MELD recipients have decreased immune competence [16] and can therefore benefit from the lowest possible doses of immunosuppressant drugs after LT. Low-dose Advagraf® with aimed trough levels of Tacrolimus at the end of the first week after LT should protect patients, especially high-MELD patients, from negative side effects of CNI, especially with regard to renal function and infection rate, while simultaneously promoting no increase in the rate of rejection. Furthermore, it is assumed that patients with a MELD-score ≤20 also benefit from this immunosuppressive regime.

The aim of this study is to evaluate the effectiveness of a low-dose Calcineurin inhibitor-based immunosuppressive regime with Advagraf® where aimed trough levels of Tacrolimus are reached at the end of the first week after liver transplantation, in regard to infectious complications and both, graft and renal function.

## Methods/Design

### Study population

The study population consists of patients scheduled for LT for any indication. Inclusion criteria are age of >18 years and <65 years, first transplantation, initial immunosuppression with Advagraf®, Corticosteroids, and Mycophenolat-mofetil (MMF) with surgery and immediate postoperative treatment in the Department of General-, Visceral- and Transplant Surgery at the University Hospital of Heidelberg.

Exclusion criteria are missing written consent, re-transplantation, and the presence of acute infections (CMV-reactivation, pneumonia, urinary tract infection, wound infection, and reactivation of hepatitis B/C). The individual criterion for discontinuation is the patient’s withdrawal of written consent.

The study protocol has been approved by the ethics committee, University of Heidelberg (S-577/2012).

### Study objectives

The primary objective is to evaluate the rate of infectious complications in the surrounding of pre-existing reduced immune competence in patients after LT under standard CNI-based immunosuppression with Advagraf®, MMF and Urbason (Table 1).

**Table 1:** Immunosuppression regime

*Urbason*	Day 0:	2 × 125 mg i.v.
	1. POD	2 × 80 mg i.v.
	2. POD	2 × 40 mg i.v.
	3. POD	2 × 20 mg i.v.
	4. POD	1 × 40 mg i.v./p.o.
*MMF*	2 × 1 g/day	Start within 12 h after LT
*Advagraf®*	1 × 0.1 mg/kg/day	Start within 12 h after LT; aimed trough level at the end of the 1st week 7-10 μg/l

i.v. = intra-venous, MMF = Mycophenolat-mofetil, p.o. = per os, POD = Postoperative day.

Patients with grafts from CMV- positive donors receive standard CMV-prophylaxis (Table 2).

**Table 2:** CMV-Prophylaxis

CMV- positive donor	Valganciclovir for 3 months:
	- 2 × 450 mg/d per os during the 1st and 2nd week after LT
	- 1 × 450 mg/d per os thereafter
	- Reduced dosages in case of renal dysfunction creatinine clearance (ml/min):
	- 40-59: 1 × 450 mg/d
	- 25-39: 450 mg every 2^nd^ day
	- 10-24: 450 mg 2x/week
	- Patients under dialysis: 450 mg 2x/week
	Ganciclovir i.v.:
	- 2x/d 5 mg/kg
	- Reduced dosages in case of renal dysfunction creatinine clearance (ml/min):
	- 49-10: 1x/d 2,5 mg/kg
	- <10: 1,25 mg/kg 2x/week
	- Patients under dialysis: 1x/d 1,25 mg/kg

As secondary parameters the correlation between the patients’ preoperative HLA-DR statuses, MELD-scores and liver functions measured by the LiMAx-Test and the correlation between the patients’ postoperative HLA-DR statuses and liver function measured by the LiMAx-Test will be assessed. Furthermore infection rate, graft- and kidney function over time will be analysed and compared between the two groups.

Clinically relevant measures of liver function (serum bilirubin, INR, serum transaminase peaks) and kidney function (creatinine, glomerular filtration rate (GFR)) as well as episodes of rejection will be documented.

### Design and trial flow

**Figure 1:**
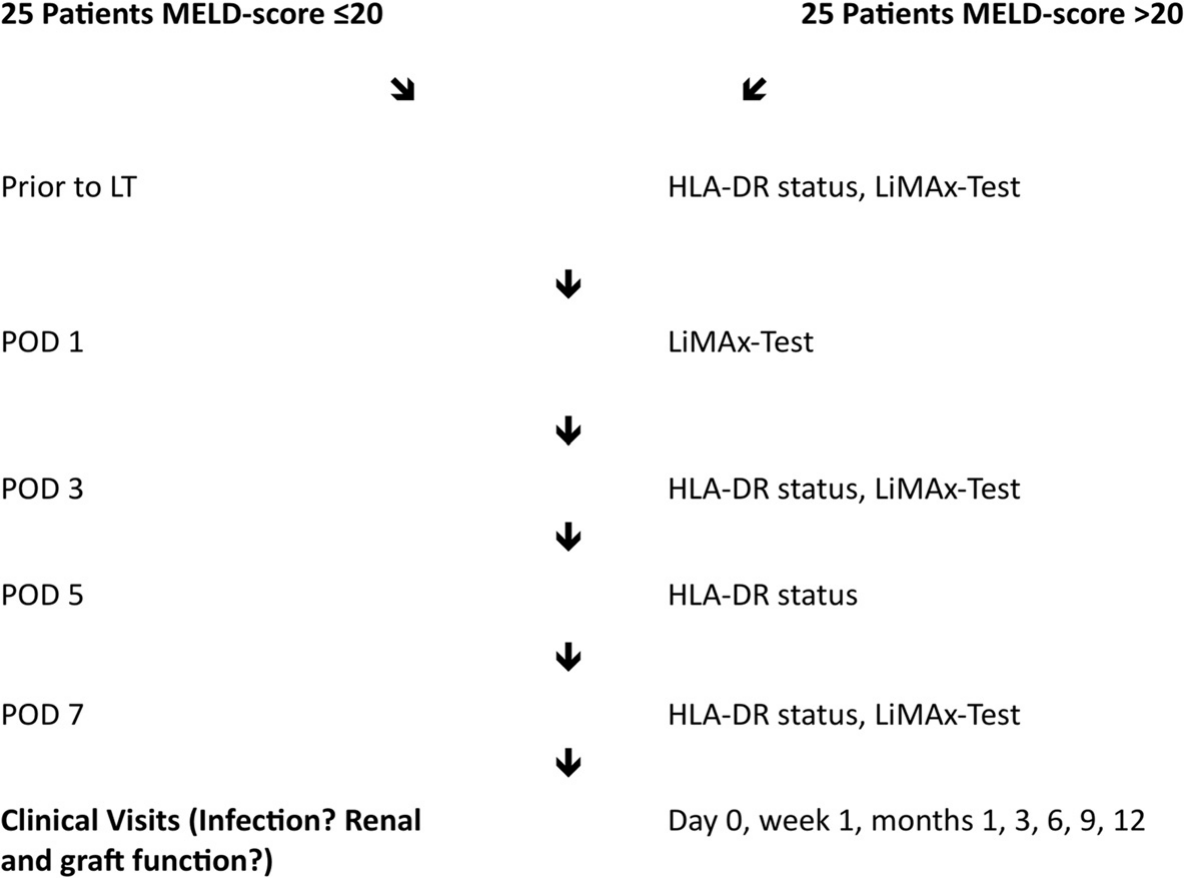
Flow chart of the IMUTECT study. HLA-DR= Human leukocyte antigen DR, LiMAx= Maximal liver function capacity based on ^13^C-Methacetin kinetics, LT= Liver transplantation, MELD= Model for end-stage liver disease, POD= Postoperative day.

For participation in this prospective, mono-centric observational study, informed written consent is mandatory. We will evaluate 50 patients, including 25 consecutive patients with a calculated pretransplant MELD-score of >20 and 25 consecutive patients with a calculated pretransplant MELD-score of ≤20. Infectious complications (CMV-reactivation, wound infection, urinary tract infection, and pneumonia), graft- and kidney function will be analysed on day 0, within the first week after LT, and 1, 3, 6, 9 and 12 months after LT.Immediately before LT and on postoperative day (POD) 3, 5, and 7, the patients’ immune status will be determined by the measurement of their HLA-DR status with the BD Quantibrite™ HLA-DR/monocyte test. The patients’ liver functions will be evaluated with the LiMAx test prior to LT and on POD 1, 3, and 7 (Figure 1).

### Sample size calculation

This is an observational pilot study. Therefore, no sample size calculation was performed. The sample size of 50 patients is resulting of the feasibility of patient recruitment within one year.

### Documentation and data handling

Protocol-required information will be documented in the case report form and reviewed and signed by the investigator or by a designated sub-investigator.

### Statistical methods

This exploratory data analysis will be performed calculating appropriate summary measures for the empirical distribution of the parameters. Correlation coefficients, such as the Spearman Coefficient, will be determined in connection with the MELD-score and all other correlations will be expressed using the Pearson Correlation Coefficient. The comparison of infection rates between the two groups will be analyzed using the chi-squared test. Continuous parameters will be analyzed using the *t*-test. Resulting p-values will be interpreted descriptively.

### Ethical issues

The trial will be performed according to the Declaration of Helsinki. Before starting the trial, the study protocol was approved by the ethics committee, University of Heidelberg. Participation is voluntary and patients can quit the trial at anytime without fear of subsequently receiving poor medical care or the need to disclose motives for withdrawal. In case of a patient’s withdrawal, the relevant data will be deleted or the patient may be asked to give consent for the analysis of the data. Patient names and all other confidential information are subject to medical confidentiality at the appointment of the German Data Protection Act. Transmission of data will be done in an encrypted format. Third party persons have no access to original documents.

Informed consent will be obtained and validated using both the physician’s and the patient’s signature.

## Discussion

The MELD-score was initially designed to estimate the prognosis of survival after TIPS [1]. Nowadays, it is one of the key scores used for assessing patients’suitability for a liver graft by focusing on the patient’s serum creatinine, bilirubin, and the INR ratios. The MELD-score values usually range between 6 and 40. Liver allocation follows the “sickest-patient-first” strategy, which has significantly decreased outcome after LT in Germany [17] and increased total costs associated with transplantation [18].

Advagraf® is a Tacrolimus derivate that must be taken once daily (in contrast to Prograf® which has to be administered twice per day). Blood levels of Advagraf® are usually increasing during the first week until the aimed Tacrolimus trough levels are reached.

There is evidence that the immune systems of high-MELD patients are compromised per se, which in turn, may lead to an increased risk of early infectious complications. Almost 85% of patients become afflicted with early infections, which is the most common cause of death soon after LT [15].

Additionally, poor renal function often occurs in high-MELD patients in the surrounding of multi-morbidity and as creatinine levels became a key parameter in the allocation of liver grafts. Early renal dysfunction after transplantation has been reported in up to 50% of patients [19,20]. CNI are well documented as major risk factors for early renal impairment and are believed to be responsible for chronic renal failure after LT [15]. Renal function seems to play a key role in the long-term survival of patients with higher MELD-scores, therefore, new immunosuppression strategies are necessary in order to avoid CNI-related impairment of the kidney function after LT.

There are several reported strategies aimed at forestalling renal impairment. One investigated method, the “Bottom-up” immunosuppression regime as described by Schnitzbauer *et al.,* documented the use of low dosages of immunosuppressants immediately after LT. The drug dosages were then increased in case of organ rejection or when patients were stable beyond day 30 [15].

In the context of the “ReSpECT” study, patients received standard doses of Tacrolimus (trough levels >10 ng/mL) and Corticosteroids, or MMF and reduced dosages of Tacrolimus (trough levels ≤8 ng/mL) and Corticosteroids, or Daclizumab induction and MMF and reduced dosages of Tacrolimus with delayed administration until the fifth day post-transplantation along with Corticosteroids. Reduced dosages of Tacrolimus have been associated with less nephrotoxicity and do not increase the risk of rejection [21]. Thus, a low-dose Advagraf®-based immunosuppression regime would decrease both the infection rate (CMV-reactivation, wound infection, urinary tract infections, and pneumonia) and associated negative side effects of a CNI-based immunosuppression strategy, especially in patients with renal impairment. Moreover, the administration of a “once-daily” formulation of Advagraf® is more “patient friendly” [22] and has similar efficacy and safety results as the “twice-daily” formulation of Tacrolimus [23].

In summary, prolonged-release, low-dose Advagraf® is predicted to better protect patients from CNI side effects when compared to standard immunosuppression regimes, while simultaneously maintaining graft function and not elevating the rate of organ rejection. It has been hypothesized that especially high-MELD recipients (MELD-score >20) who have decreased immune competence and a reduced renal function benefit from low-dose Advagraf® regime in regard to infectious complications and renal function. Furthermore, it can be assumed that every patient would benefit from the clinical application of this strategy after LT.

We have chosen a prospective observational study design with a minor sample size as randomization is not possible in regard to our study objectives. Depending on the results of this study, a randomized controlled trial with adjusted study objectives and a major sample size might be useful in following study protocols.

## Trial status

### Recruiting

Enrolment of the first patient was performed in June 2013.

## Abbreviations

CMV: Cytomegalie virus; CNI: Calcineurin inhibitor; CYP: Cytochrome P450; DIC: Disseminated intravascular coagulation; EDTA: Ethylene diaminetetraacetic acid; GFR: Glomerular filtration rate; GM-CSF: Granulocyte-Macrophage Colony-Stimulating Factor; HLA: Human leukocyte antigen; INR: International normalized ratio; LiMAx: Maximal liver function capacity based on ^13^C-Methacetin kinetics; LT: Liver transplantation; MELD: Model for end-stage liver disease; MHC: Major histocompatibility complex; MMF: Mycophenolat-mofetil; POD: Postoperative day; TIPS: Transjugular intrahepatic porto-systemic shunt

## Competing interests

This study was supported by an unrestricted grant from Astellas Pharma, Munich, Germany. Astellas was not involved in the design of the study, in the decision to submit the manuscript for publication or in the writing of the manuscript.

## Authors’ contributions

SR and PS designed the study and drafted the manuscript. MD, PH, GP, DG, CDH and TG conceptualized the study and participated in its design and coordination. AS participated in the design of the study and the statistical analysis. These authors also assisted in drafting the manuscript. All authors read and approved the final manuscript.

## Pre-publication history

The pre-publication history for this paper can be accessed here:

http://www.biomedcentral.com/1471-2482/14/64/prepub
